# Ischemic stroke risk estimation in patients without oral anticoagulation: an observational cohort study based on secondary data from Germany

**DOI:** 10.1186/s12872-019-1074-7

**Published:** 2019-04-23

**Authors:** Felix S. Wicke, Martin A. Schaller, Kateryna Karymova, Martin Beyer, Beate S. Müller

**Affiliations:** 1 Institute of General Practice, Goethe-University, Theodor-Stern-Kai 7, 60590 Frankfurt, Germany; 2 Department of Neurology, Goethe-University, Schleusenweg 2-16, 60528 Frankfurt, Germany

**Keywords:** Atrial fibrillation, Stroke, Ischemia, CHA_2_DS_2_-VASc-score, Anticoagulation, Risk assessment, Cardiovascular epidemiology, Incidence rate

## Abstract

**Background:**

Oral anticoagulants can cause potentially serious adverse events. Therefore, before prescribing oral anticoagulants for ischemic stroke prevention in patients with atrial fibrillation (AF), stroke risk assessment is required to identify patients that are likely to benefit from treatment. Current guidelines recommend the CHA_2_DS_2_-VASc-score for stroke risk assessment. The CHA_2_DS_2_-VASc-score is based on observational studies from different treatment settings and countries. As ischemic stroke risk differs by setting and region, the aim of this study is to estimate ischemic stroke risk (stratified by the CHA_2_DS_2_-VASc-score) for a broadly representative population with AF from southern Germany and compare them to results from previous studies.

**Methods:**

The study design is a retrospective cohort study on patients with atrial fibrillation based on secondary data. We calculated CHA_2_DS_2_-VASc-score based on patient’s diagnoses recorded in the year 2014 and assessed outcomes in 2015–2016. The primary outcome is hospitalization for ischemic stroke. The secondary outome is hospitalizations for any thromboembolic event, including ischemic stroke, transient ischemic attack, peripheral arterial embolism, pulmonary embolism, and mesenterial embolism. We estimated the incidence rates of the outcomes (and corresponding 95%-confidence intervals) stratified by CHA_2_DS_2_-VASc-score.

**Results:**

The primary endpoint occurred in 961 of the 30,299 patients constituting the study population, resulting in a total incidence rate of 2.2 per 100 person-years. The secondary endpoint occurred in 1553 patients (3.6 per 100 person-years). Ischemic stroke rates stratified by the CHA_2_DS_2_-VASc-score tended to be lower than those reported previously. Thromboembolic event rates stratified tended to be similar to those reported previously.

**Conclusions:**

Our results show that the performance of the CHA_2_DS_2_-VASc-score differs in the German population, as compared to internationally published data, with an overall trend towards lower risk of ischemic stroke in uncoagulated patients with AF. These results should not be practice changing, but they emphasize that stroke risk estimation in patients with atrial fibrillation should be further refined.

## Background

Results from randomized controlled trials (RCTs) clearly demonstrate the benefits of oral anticoagulation in patients with atrial fibrillation (AF) [1, 2]. Whereas most patients are likely to benefit from oral anticoagulation by prevention of ischemic strokes, some patients will suffer from bleeding complications due to anticoagulation. Treatment with oral anticoagulants therefore requires a careful risk-benefit assessment.

Evidence-based estimates that help to identify which patients are more likely to suffer from bleeding events than to benefit from prevention of thromboembolism are needed. Results from randomized and controlled trials (RCTs) would be ideal, because they are not prone to confounding by indication or other biases. However, available RCTs are not powered to identify valid high- or low-risk characteristics; instead, observational studies are used to identify such characteristics. Guidelines recommend ischemic stroke risk assessment based on the CHA_2_DS_2_-VASc-score [3–5]. Current guideline recommendations are to start oral anticoagulation for men with a score of 2 or higher and for women with a score of 3 or higher, if there is no excessively high bleeding risk. Oral anticoagulation should be considered for male patients with CHA_2_DS_2_-VASc-score of 1 and for female patients with a score of 2 [3–5].

Multiple observational studies have been conducted to estimate risk of thromboembolism stratified by the CHA_2_DS_2_-VASc-score in patients with uncoagulated AF [6–12]. These studies have differed in the population they included: some used specific trial populations and others used inpatient registries or national databases. As patients from different levels of care (eg. general practice versus specialist care) are likely to differ in their risk for ischemic events, the selected population is of great importance for generalizability and interpretation. Accordingly, a systematic review of the topic reported wide variation in incidence rates of ischemic stroke in patients with AF [13].

Clinical implications for the use of the CHA_2_DS_2_-VASc-score would potentially arise, if incidence rates can be shown to differ not only between levels of care, but also geographically (e.g. between countries). To our knowledge, no study reported data for ischemic stroke risk prediction for patients with AF from Germany. Therefore, the aim of this study is to estimate ischemic stroke risk (stratified by the CHA_2_DS_2_-VASc-Score) for a broadly representative population with AF from southern Germany.

## Methods

### Study design

This is a retrospective cohort study with a two-year observation period (2015–2016) on patients with AF based on secondary data.

### Data source

Claims data from a statutory health insurance (AOK Baden Wuerttemberg), the largest insurance fund in the German state of Baden-Wuerttemberg (population in 2014 was 10.7 million), were used. For the year 2014, the data contains information on 3.8 million individuals, which equals to about 35% of the state’s population. The dataset contains information on all insured persons, including age, sex, hospital and outpatient treatments with respective diagnoses (coded according to the International Classification of Disease Version 10, ICD-10), and all outpatient drug prescriptions (coded according to the Anatomical-Therapeutic-Chemical Classification, ATC).

### Study population

From the total dataset we identified all patients aged 18 years or older with a diagnosis of AF recorded in 2014. To increase diagnostic specificity, we required outpatient diagnoses to be coded in at least two quarters of the year 2014. For hospital diagnoses, only one coding was required. We excluded patients with coded rheumatic mitral valve disease or artificial heart valves and those that died in 2014. The study cohort consists of all patients with AF and without oral anticoagulation in 2014. We identified oral anticoagulants based on ATC codes of prescription data, including vitamin K antagonists (ATC: B01AA), direct factor Xa inhibitors (B01AF) and Dabigatran (B01AE07).

### Variables

We used ICD-10 coded diagnoses of the year 2014 for the calculation of the CHA_2_DS_2_-VASc-score. In- and outpatient diagnoses were used and the CHA_2_DS_2_-VASc-score was calculated as described in Table 1.

**Table 1:** CHA_2_DS_2_-VASc-score

	Risk Factor	Points
C	Congestive heart failure	1
H	Hypertension	1
A_2_	Age ≥ 75 years	2
D	Diabetes mellitus	1
S_2_	Prior Stroke or transient ischemic attac	2
V	Vascular disease	1
A	Age 65 to 74 years	1
Sc	Sex category (female)	1
	Range of possible scores	0 to 9

For the outcome assessment we recorded all hospitalizations for ischemic stroke, further thromboembolic events (peripheral emboli, pulmonary emboli and transient ischemic attack) and deaths in 2015 and 2016 (ICD codes see below). To exclude the possibility that a diagnosis of AF was related to an acute ischemic event, we used a ‘blanking period’ of 14 days (as suggested by Friberg and colleagues [7]) between baseline and follow-up assessment.

The primary outcome was hospitalization for ischemic stroke. We recorded all hospitalizations with ischemic stroke (ICD-10 code I63) as primary discharge diagnosis and the respective hospital admission date. The secondary outcome was hospitalization for any thromboembolic event (TE; alternative terminology: stroke and systemic embolism). TE includes ischemic stroke (I63), transient ischemic attack (G45), peripheral arterial embolism (I74), pulmonary embolism (I26) and mesenterial embolism (K55).

For patients with multiple occurrences of the outcome, only the first was counted, as later re-hospitalizations are often related to the first event. For calculation of person-time at risk, we ended individual follow-up for patients at time of the outcome of interest, death, beginning of treatment with oral anticoagulation, or dropout from the dataset (e.g. due to change of insurance company).

Because of the observational study design, patients might not have received oral anticoagulation due to excessive risk of bleeding. Therefore, we provide estimates of bleeding rates to allow a more detailed assessment of the cohort. We included hospitalizations due to intracranial bleeding (I60, I61, I62, gastrointestinal bleeding (I85.0, I98.3, K25.0, K25.2, K25.4, K25.6, K26.0, K26.2, K26.4, K26.6, K27.0, K27.2, K27.4, K27.6, K28.0, K28.2, K28.4, K28.6, K62.5, K92.2) and acute posthemorrhagic anemia (D62).

### Statistical methods

We calculated incidence rates and 95%-confidence intervals for primary and secondary outcomes, stratified by the CHA_2_DS_2_-VASc-score. To assess the predictive performance of the risk score, we calculated the concordance index (c-index) of the scores for prediction of ischemic stroke events as area under the curve of corresponding receiver operating characteristic (ROC)-curves [14]. All analyses were done using R (version 3.4.2) [15] and the R package ‘pROC’ [16].

### Sensitivity analysis

Because the diagnostic certainty of AF and ischemic stroke rates could be different depending on different health care settings, we calculated incidence rates of ischemic stroke (with 95%-confidence intervals, stratified by CHA_2_DS_2_-VASc-score) for patients that had a coded diagnosis of AF only from the outpatient setting, only from the inpatient setting, and for those with in- and outpatient diagnosis. In addition, we calculated incidence rates for those with specifically coded diagnosis of permanent atrial fibrillation (I48.2).

## Results

From the total dataset of 3.81 million insured persons, we identified 107,777 patients with nonvalvular AF (approximate prevalence of 2.8%) in 2014. Of those, 30,229 patients (28.0%) did not receive oral anticoagulation in 2014 (the study population). Their baseline characteristics are summarized in Table 2. For comparison, we also provide in Table 2 baseline characteristics of patients who received at least one prescription of an oral anticoagulant in 2014. Patients with oral anticoagulation have a slightly higher CHA_2_DS_2_-VASc-score and more comorbidities fitting a cardiovascular risk profile (e.g. hypertension, diabetes, vascular disorders).

**Table 2:** Baseline characteristics of patients with atrial fibrillation with and without oral anticoagulation in 2014

	With OAC (*N* = 82,331)	Without OAC (*N* = 30,229)
Age (mean ± SD)	77.12 ± 8.75	76.41 ± 12.42
Sex (percent male)	47.7%	46.6%
CHA_2_DS_2_-VASc-score (mean ± SD)	4.66 ± 1.57	4.25 ± 1.81
Hypertension	91.6%	85.0%
Chronic heart failure	47.7%	40.2%
Stroke or transient ischemic attack in 2014	10.1%	7.96%
Stroke in 2014	6.4%	4.71%
Diabetes mellitus	44.3%	10.1%
Vascular disease	50.6%	12.0%
Alcohol-related disorder	3.2%	5.2%
Dementia	11.3%	19.2%
Anemia	16.2%	19.9%
Renal disease	32.8%	31.90%
Dialysis	0.9%	2.5%
Liver disease	15.7%	15.0%

*OAC* oral anticoagulation

The primary endpoint (ischemic stroke) occurred in 961 of the 30,299 patients constituting the study population, resulting in an incidence rate of 2.2 per 100 person-years. The secondary endpoint (thromboembolic events) occurred in 1553 patients (3.6 per 100 person-years). Incidence rates with 95%-confidence intervals for ischemic strokes and thromboembolic events stratified by CHA_2_DS_2_-VASc-score are shown in Table 3. The risk of ischemic stroke and thromboembolism clearly increases with increasing CHA_2_DS_2_-VASc-score and, as judged by 95-% confidence intervals, the score discriminates sufficiently between different levels of risk. The c-index for the primary outcome was 0.608.

**Table 3:** Incidence rates of ischemic stroke, thromboembolic events and bleeding events stratified by CHA_2_DS_2_-VASc-score in patients with atrial fibrillation and without oral anticoagulation (N = 30,229)

CHA_2_DS_2_-VASc	N	ischemic strokes				Thrombembolic events				Bleeding			
		events	incidence rate (per 100py)	95%-CI		events	incidence rate (per 100py)	95%-CI		events	incidence rate (per 100py)	95%-CI	
0	721	1	0.08	0.01	0.55	4	0.31	0.12	0.82	2	0.15	0.04	0.62
1	1881	16	0.48	0.30	0.79	26	0.79	0.54	1.16	11	0.33	0.19	0.60
2	2636	47	1.08	0.82	1.44	83	1.92	1.55	2.37	21	0.48	0.32	0.74
3	4208	85	1.30	1.05	1.60	137	2.10	1.78	2.47	34	0.52	0.37	0.72
4	6394	215	2.31	2.02	2.63	332	3.58	3.22	3.98	84	0.90	0.73	1.11
5	6784	243	2.58	2.28	2.93	390	4.18	3.79	4.60	87	0.92	0.75	1.14
6	4823	200	3.27	2.86	3.75	341	5.62	5.07	6.24	78	1.28	1.02	1.59
7	2066	107	4.18	3.47	5.04	164	6.44	5.55	7.46	31	1.21	0.85	1.71
8	530	33	5.31	3.81	7.39	53	8.58	6.64	11.10	10	1.59	0.86	2.93
9	186	14	7.22	4.36	11.96	23	12.27	8.37	18.0	4	2.03	0.77	5.35
Total	30,229	961	2.20	2.07	2.34	1553	3.55	3.38	3.73	362	0.83	0.75	0.92

Thromboembolic events include ischemic stroke, transient ischemic attack, peripheral arterial embolism, pulmonary embolism, and mesenterial embolism

Bleeding events include intracranial bleeding, gastrointestinal bleeding and acute posthemorrhagic anemia

*CI* confidence interval, *py* person-years

The estimated incidence rate hospitalization due to a bleeding event was 0.83 per 100 person-years (compare Table 3). This rate is low in comparison to published rates from similar studies [7, 17], definitely ruling out an excessive bleeding risk, on average, in this study’s cohort.

**Fig. 1:**
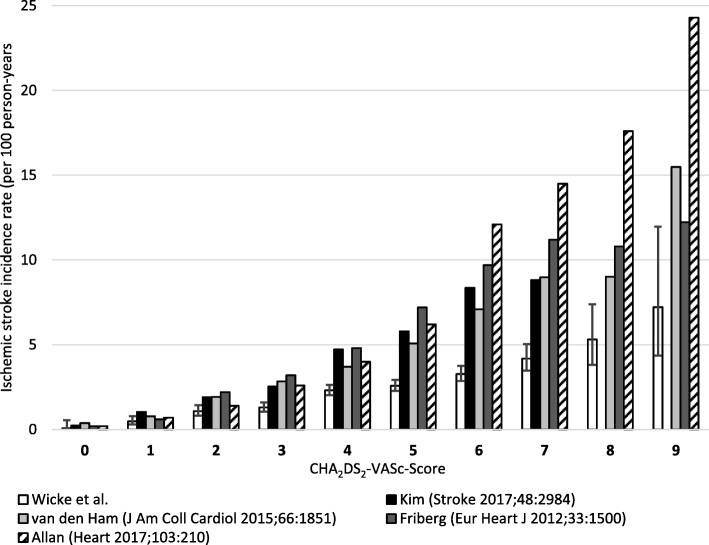
Comparison of ischemic stroke incidence rates in patients with atrial fibrillation and without oral anticoagulation (whiskers show 95%-confidence intervals)

A comparison of estimated incidence rates with those reported in the literature is shown in Table 4 and Fig. 1. Ischemic stroke rates stratified by the CHA_2_DS_2_-VASc-score are lower than those reported by Friberg [7], van den Ham [8], Kim [9] and Allan [12]. Thromboembolic event rates stratified by CHA_2_DS_2_-VASc-score approximately fall between those reported by Olesen [11] and Singer [10]. Especially with higher CHA_2_DS_2_-VASc-scores (≥5), estimates from this study are considerably lower. However, there is large variability in reported rates.

**Table 4:** Comparison of reported incidence rates of ischemic stroke (IS) and thromboembolic events (TE) stratified by CHA_2_DS_2_-VASc-score

CHA_2_DS_2_-VASc	Wicke et al.		Lip (6)	Friberg (7)		van den Ham (8)	Kim (9)	Singer (10)	Olesen (11)	Allan (12)
	IS	TE	TE	IS	TE	IS	IS	TE	TE	IS
0	0.1	0.3	0	0.2	0.3	0.4	0.2	0.0	0.7	0.2
1	0.5	0.8	0.6	0.6	0.9	0.8	1.0	0.6	1.5	0.7
2	1.1	1.9	1.6	2.2	2.9	1.9	1.9	0.8	2.9	1.4
3	1.3	2.1	3.9	3.2	4.6	2.8	2.5	1.7	4.3	2.6
4	2.3	3.6	1.9	4.8	6.7	3.7	4.7	2.8	6.5	4.0
5	2.6	4.2	3.2	7.2	10	5.1	5.8	4.3	10.0	6.2
6	3.3	5.6	3.6	9.7	13.6	7.1	8.4	4.8	12.5	12.1
7	4.2	6.4	8.0	11.2	15.7	9.0	8.8	4.8	14.0	14.5
8	5.3	8.6	11.1	10.8	15.2	9.0		7.8	14.1	17.6
9	7.2	12.3	100	12.2	17.4	15.5		16.6	15.9	24.3
Total	2.2	3.6	~ 2.4^a^	4.5	6.2	2.9	4.0	2.1	n/a^b^	3.8

^a^Total incidence rate not given in original publication, but approximated from given data

^b^Total incidence rate not available from original publication

The results of the sensitivity analysis (Table 5) show a trend towards higher incidence rates for those patients with in- and outpatient diagnoses of AF and those with permanent AF, albeit with largely overlapping 95%-confidence intervals.

**Table 5:** Sensitivity analysis showing ischemic stroke incidence rates stratified by CHA_2_DS_2_-VASc-score for those patients with coded diagnoses of atrial fibrillation only from the outpatient, only from inpatient and in- & outpatient settings

CHA_2_DS_2_-VASc	ischemic stroke incidence rates (per 100 person-years) [95%-confidence intervals]			
	outpatient diagnosis *N* = 18,915	inpatient diagnosis *N* = 6887	in- and outpatient diagnosis *N* = 4427	only permanent atrial fibrillation (ICD-10: I48.2) *N* = 5033
0	0.11 [0.02–0.76]	0	0	0
1	0.44 [0.24–0.79]	0.82 [0.31–2.16]	0.35 [0.05–2.47]	1.47 [0.48–4.51
2	1.18 [0.86–1.61]	0.57 [0.22–1.52]	1.25 [0.47–3.32]	0.94 [0.31–2.91]
3	1.24 [0.97–1.59]	1.61 [1.02–2.55]	1.15 [0.52–2.55]	1.87 [1.11–3.13]
4	2.26 [1.93–2.65]	2.26 [1.66–3.06]	2.70 [1.85–3.95]	3.83 [2.90–5.06]
5	2.39 [2.03–2.81]	2.39 [1.83–3.13]	3.80 [2.89–5.01]	2.84 [2.13–3.77]
6	2.67 [2.17–3.28]	3.50 [2.72–4.51]	4.69 [3.61–6.08]	3.99 [3.04–3.77]
7	3.68 [2.75–4.92]	3.99 [2.84–5.60]	5.54 [3.94–7.80]	5.25 [3.70–7.43]
8	4.86 [2.73–8.65]	4.25 [2.32–7.80]	7.48 [4.34–12.89]	5.47 [2.79–10.74]
9	5.38 [2.08–13.96]	8.07 [3.48–18.71]	8.67 [3.74–20.05]	6.63 [2.57–17.08]

## Discussion

### Key results

Our results show that incidence rates of ischemic stroke in uncoagulated patients with AF are lower in Germany compared to internationally reported incidence rates.

Most of those studies report either rates of ischemic strokes or rates of thromboembolic events (including peripheral arterial embolism and pulmonary embolism in addition to cerebral ischemia). This limits comparability of results. We believe that ischemic stroke is the most relevant outcome, as it is clearly linked to AF. We agree with Friberg et al. [18] that transient ischemic attack, which by definition has a benign outcome, is not a good outcome for studying stroke risk in this context. The rationale for including pulmonary embolism as an outcome is that it is potentially preventable by oral anticoagulation. Whether AF itself is a relevant causative risk factor for pulmonary embolism is under debate [19–22], but in general, pulmonary emboli are thought to arise mainly from thrombosis in the lower extremities [23]. We decided to use ischemic strokes as primary outcome and report thromboembolic events as secondary outcome.

Incidence rate estimates for ischemic strokes from this study are lower than previously reported estimates, but those for thromboembolic events are within the range of previous studies. This suggests the possibility that the outcomes stroke and thromboembolic events might be detected or recorded differently in different health-care settings (e.g. stroke as transient ischemic attack). We suggest that studies on the topic should optimally report both outcomes.

There are multiple methodological challenges in estimating risks in patients with AF: RCTs have shown the overall advantage of anticoagulation, but have been not been powered or designed in regard to risk-benefit stratification based on baseline characteristics, which are required for individual clinical decision making. Therefore risk estimation relies on observational studies of patients with and without anticoagulation, which suffers from potential confounding by indication [24], that is, patients actually receiving anticoagulation are possibly (and likely) different in key characteristics related to ischemic stroke and bleeding risks from those that do not receive anticoagulation. These differences can result from physician’s or patient’s decisions for or against anticoagulation and may be based on false beliefs regarding oral anticoagulants (e.g. overestimating the risk of falling or underestimating benefits of anticoagulation in elderly patients).

### Limitations

Confounding by indication is a possible source of bias in this study and our data does not contain information on the specific reasons for or against anticoagulation in the cohort. One aspect of confounding by indication is bleeding risk, either objective or subjective fear of bleeding by either patient or physician, which could account for the decision against oral anticoagulation. Bleeding incidence for this study’s cohort, however, are not excessive (Table 3) and we thus conclude that objective bleeding risk is not a major confounding factor.

Further limitations of the study are the potential for exposure, covariate and outcome misclassification inherent to secondary data analysis. Exposure misclassification regarding AF is possible, as we rely on the validity of coded diagnoses and had no possibility of electrocardiogram-based verification. We included codes for paroxysmal and persistent AF and for atrial flutter, because all diagnoses are indications for oral anticoagulation [3]. The prevalence estimate of AF in our study is slightly higher than previous results from Germany: 2.5% was reported from the Gutenberg-Health Study [25] and Wilke et al. [26] reported 2.1% (but they included persistent AF, only). To increase validity of AF diagnoses, we required a diagnostic code from two different quarters in the baseline year 2014. Additionally we conducted a sensitivity analysis, to check whether incidence rates of ischemic strokes differ for patients with AF diagnoses exclusively from the outpatient, inpatient and in- and outpatient setting (Table 5). This analysis shows a trend towards higher incidence rates for patients with diagnostic codes from the in- and outpatient setting. Possible explanations are that there is some degree of overcoding (e.g. false-positive diagnoses) or that the trend reflects the higher risk of patients that were previously hospitalized, but the sensitivity analysis does not explain the overall comparatively low incidence rates. Also, inclusion of unspecific and paroxysmal AF does not seem to explain the comparatively low ischemic stroke rates, as estimates for the subgroup with permanent AF largely overlap with the total cohort (Table 5).

Covariates, specifically comorbidities used for the calculation of the CHA_2_DS_2_-VASc-score, were assessed based on the year 2014 only. Therefore, it is possible that existing conditions, like a stroke or transient ischemic attack that occurred in the years before, could be missed in the calculation of the score. This would result in an underestimation of the true score and in turn to an overestimation of stroke risk in the corresponding score group.

Germany has a well-established acute care system for stroke, including nearly universal access to specialized stroke units. The vast majority of hospitalized patients with suspected stroke receive neuroimaging. We therefore consider the validity of the primary outcome as high. The ICD-10, however, does not allow coding of the exact etiology of embolic strokes. The primary outcome thus includes cases of non-cardioembolic ischemic strokes (e.g. embolism from aortic, cervical or cranial plaques or microangiopathic ischemia), which is a relevant concern in studies of risk estimation in AF, because the preventive treatment of choice for atherosclerotic thromboembolism is thrombocyte aggregation inhibition. We could not account for ischemic strokes that did not lead to hospital admission, e.g. because patients died or did not seek care.

Estimates for those with low risk of events and sensitivity analyses are limited by relatively wide confidence intervals, despite the relatively large cohort size.

### Interpretation

Our results suggest that ischemic stroke incidence rates are lower for the observed population from southern Germany in comparison to internationally reported rates. In addition to the limitations discussed above, we give possible explanations for this observation.

Risk compositions are likely to be different in AF cohorts sampled from hospital registries or specialist care and those sampled from primary care or the general population, as was shown by Allan et al. [12] for patients from Great Britain. Friberg et al. [7] used data from a Swedish hospital discharge registry, which might not be representative for primary care or the general population. Kim et al. [9] used data from the Korean National Health Insurance, but risks are probably different in Korean and European populations.

The risk of ischemic stroke might change over time. Our recent data (2015 and 2016) could reflect those trends in comparison to previous studies, which used data sources going back into the 1990s and epidemiological studies show a trend toward declining overall ischemic stroke incidence rates [27]. This could be part of the explanation for the comparatively low rates observed in this study. Increased awareness of physicians and patients of AF could lead to earlier detection, at a stage where risk of thromboembolism might be lower. Detection rates could furthermore be different depending on characteristics of the health care system (e.g. number of physician visits or health “check-ups”), which in turn could influence the risk composition. Also, optimized treatment of comorbidities might lower the relative risk associated with each specific comorbidity used for calculation of the CHA_2_DS_2_-VASc-score.

Geographic variation in anticoagulation treatment could change the risk composition of the uncoagulated populations. Wide geographic variation in oral anticoagulation has been observed [28, 29]. In regions with high coverage of oral anticoagulation, this would leave a relatively low risk composition in the uncoagulated population and vice versa.

We reported incidence rates based on the first event occurring in each patient, because additional hospitalizations have a high likelihood of being related to the first. However, this does not need to be the case and a different approach would be to count further events for the calculation of incidence rates (optimally with a latency period between events). This, of course, would result in higher incidence rate estimates. Unfortunately, most published studies do not specify how they treated multiple events, which limits comparability.

### Generalizability

In Germany, statutory health insurance covers about 90% of the population; most of the remainder are high-income and privately insured patients. Overall, we consider the cohort therefore as fairly representative of the patient population in German practices and hospitals.

## Conclusion

Our results suggest that the performance of the CHA_2_DS_2_-VASc-score differs in the German population compared to internationally published data, with an overall trend towards lower risk of ischemic stroke in uncoagulated patients with AF. Because of the diverse methodological challenges these results should at this stage not yet be practice changing. Rather we believe that stroke risk estimation in patients with atrial fibrillation should be further refined. This could be done with epidemiological studies ideally taking into account factors more specific to individual risk (e.g. echocardiography [30, 31] and biomarkers [32]) and with specific outcome assessment (only counting ischemia likely to be of cardioembolic origin).

## Abbreviations

AF: Atrial fibrillation
ATC: Anatomical-therapeutic-chemical classification
CI: Confidence interval
C-Index: Concordance index
ICD-10: International classification of disease, version 10
OAC: Oral anticoagulation
RCT: Randomized and controlled trial
ROC: Receiver operating characteristic
TE: Thromboembolic event
